# Natural polymorphisms in *ZMET2* encoding a DNA methyltransferase modulate the number of husk layers in maize

**DOI:** 10.1093/plphys/kiae113

**Published:** 2024-03-02

**Authors:** Zi Wang, Aiai Xia, Qi Wang, Zhenhai Cui, Ming Lu, Yusheng Ye, Yanbo Wang, Yan He

**Affiliations:** State Key Laboratory of Maize Bio-breeding, National Maize Improvement Center of China, China Agricultural University, Beijing 100094, China; State Key Laboratory of Maize Bio-breeding, National Maize Improvement Center of China, China Agricultural University, Beijing 100094, China; State Key Laboratory of Maize Bio-breeding, National Maize Improvement Center of China, China Agricultural University, Beijing 100094, China; Shenyang Key Laboratory of Maize Genomic Selection Breeding, Shenyang Agricultural University, Shenyang 110866, China; Maize Research Institute, Jilin Academy of Agricultural Sciences, Gongzhuling 136100, China; Maize Research Institute, Liaoning Academy of Agricultural Sciences, Shenyang 110065, China; Maize Research Institute, Liaoning Academy of Agricultural Sciences, Shenyang 110065, China; State Key Laboratory of Maize Bio-breeding, National Maize Improvement Center of China, China Agricultural University, Beijing 100094, China

## Abstract

DNA methylation affects agronomic traits and the environmental adaptability of crops, but the natural polymorphisms in DNA methylation–related genes and their contributions to phenotypic variation in maize (*Zea mays*) remain elusive. Here, we show that a polymorphic 10-bp insertion/deletion variant in the 3′UTR of *Zea methyltransferase2* (*ZMET2*) alters its transcript level and accounts for variation in the number of maize husk layers. *ZMET2* encodes a chromomethylase and is required for maintaining genome-wide DNA methylation in the CHG sequence context. Disruption of *ZMET2* increased the number of husk layers and resulted in thousands of differentially methylated regions, a proportion of which were also distinguishable in natural *ZMET2* alleles. Population genetic analyses indicated that *ZMET2* was a target of selection and might play a role in the spread of maize from tropical to temperate regions. Our results provide important insights into the natural variation of *ZMET2* that confers both global and locus-specific effects on DNA methylation, which contribute to phenotypic diversity in maize.

## Teaser

Natural variation in *Zea methyltransferase2* confers both global and locus-specific effects on DNA methylation, thereby contributing to phenotypic diversity in maize.

## Introduction

Epigenetic modifications are heritable chemical changes that do not alter the DNA nucleotide sequence but can influence the phenotype of an organism (Lucibelli et al. 2022). Epigenetics is a fascinating topic in plant biology because DNA methylation has also been suggested to be involved during development, in the response to environmental stresses and during other processes (Matzke and Mosher 2014; Zhang et al. 2018; Liu et al. 2022). Plant DNA methylation occurs through the linkage of a methyl group (–CH_3_) to cytosine in symmetric, CG and CHG, and asymmetric, CHH, contexts (in which “H” is any nucleotide except “G”) and is carried out by a family of enzymes called DNA methyltransferases (Law and Jacobsen 2010; Zhang et al. 2018; Liu et al. 2022). DNA methyltransferases are sorted into 3 categories based on their protein structure and function: methyltransferase 1 (MET1), chromomethylase (CMT), and domains rearranged methyltransferase (DRM). MET1 maintains DNA methylation of CG during DNA replication (Kankel et al. 2003; He et al. 2011). CMT, which is unique to plants, maintains DNA methylation of CHG and CHH and maintains heterochromatin status (Lindroth et al. 2001; Stroud et al. 2014). DRM carries out de novo methylation of asymmetric CHH sequences, which is mediated by RNA-directed DNA methylation, a plant-specific RNA silencing pathway directed by 24-nt small interfering RNAs (Huettel et al. 2006; Zemach et al. 2013; Liu et al. 2014; Erdmann and Picard 2020). The actual DNA methylation patterns and levels are balanced by the activity of both DNA methyltransferase and DNA demethylase. In plants, DNA methylation can be removed by the family of DNA glycosylase enzymes, which include DEMETER and REPRESSOR OF SILENCING 1 (ROS1) (Zhang and Zhu 2012; Wu and Zhang 2017; Xu et al. 2022).

Our understanding of molecular mechanisms establishing DNA methylation patterns in plants is largely derived from studies in *Arabidopsis* (*Arabidopsis thaliana*); however, tremendous genomic differences exist between *Arabidopsis* and other plants, including maize (*Zea mays*). The maize genome is larger than that of *Arabidopsis* (2,500 versus 125 Mb) and contains substantially more transposable elements (Baucom et al. 2009; Schnable et al. 2009). Whereas most *Arabidopsis* genes are adjacent to other genes, many maize genes are flanked by heavily methylated transposons (Jiao et al. 2017; Stitzer et al. 2021). There are also several differences in the genome-wide distribution of DNA methylation in maize relative to *Arabidopsis*. Maize contains higher levels of CG and CHG methylation but lower levels of CHH methylation than *Arabidopsis* (Zemach et al. 2013; Bewick et al. 2017; Fu et al. 2018; Long et al. 2019). CG methylation and CHG methylation are commonly found in maize transposons but are found less frequently near genes. In contrast, CHH methylation tends to occur upstream of the transcription start site of maize genes and is associated only with certain types of transposons (Gent et al. 2013). A total of 7 DNA methyltransferase–related genes have been identified in maize, including 2 *MET1*-like genes (*ZMET1a* and *ZMET1b*), 2 genes that encode CMT family CMTs (*ZMET2* and *ZMET5*), and 4 *DRM*-like genes (*ZMET3*, *ZMET6*, and *ZMET7*) (Candaele et al. 2014; Li et al. 2014; Bewick et al. 2017). In addition, the maize genome contains 4 genes that encode DNA demethylases, which are *ZmROS1a*, *ZmROS1b*, *ZmROS1c*, and *ZmROS1d* (Xu et al. 2022).

DNA methylation levels and patterns vary across different individuals in the same species (Eichten et al. 2013; Kawakatsu et al. 2016). Natural polymorphisms in DNA methylation components that contribute to phenotypic variation have been identified in *Arabidopsis Variant in Methylation 1* (Woo et al. 2007), *CMT2*, and *NRPE1* (Kawakatsu et al. 2016; Sasaki et al. 2019) and in rice (*Oryza sativa*) *CLSY1* (Castano-Duque et al. 2021). However, we still lack the fundamental knowledge about whether genetic polymorphisms in DNA methylation–related genes are responsible for natural variation in maize growth and development.

In this study, we identified a 10-bp insertion/deletion (Indel) in the 3′UTR of *ZMET2* that is the causative polymorphism for natural variation in the number of husk layers. *ZMET2* mediates the genome-wide pattern of CHG and CHH methylation, and its genetic polymorphisms likely confer natural variation in DNA methylation at thousands of genomic sites, which in turn influences the transcription of genes involved in husk development. Evolutionary analysis revealed that the Indel-7080Ref allele associated with fewer husk layers was targeted by selection and became nearly fixed in maize temperate inbred lines, suggesting that *ZMET2* might contribute to a beneficial advantage for maize growth in maize domestication and adaptation.

## Results

### Indel-7080 within the 3′UTR of *ZMET2* contributes to natural variation in husk layer numbers

**Figure 1.**
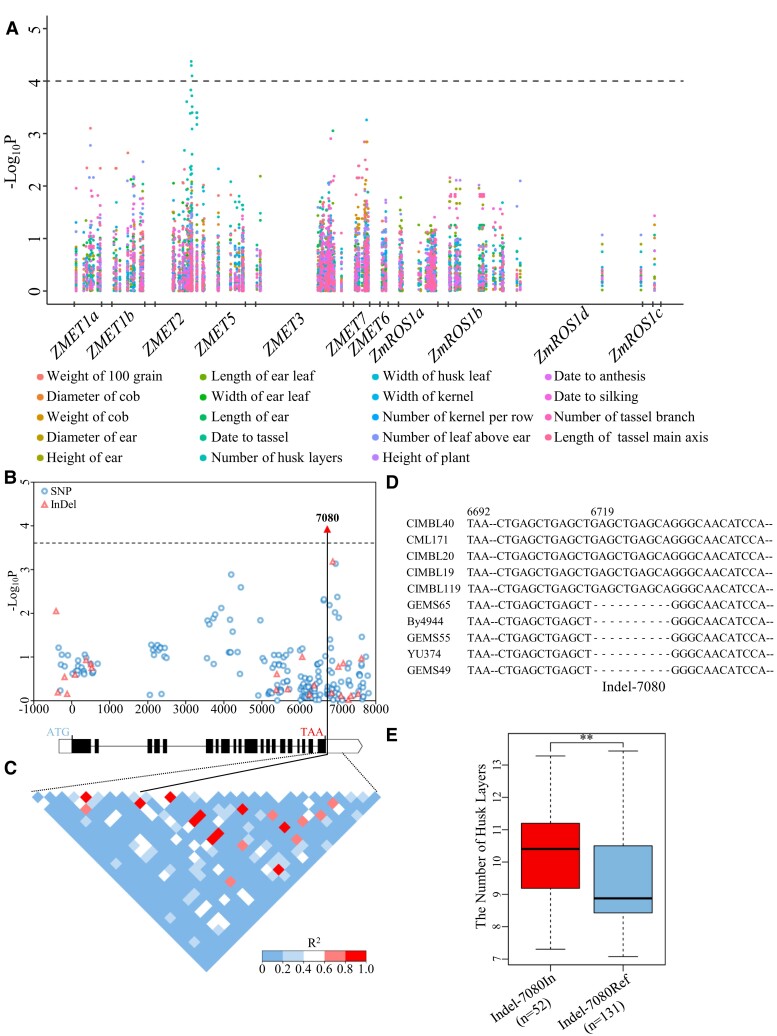
Association analysis of genetic variations in *ZMET2* with the number of husk layer. **A)** Association analysis of genetic polymorphisms in 7 DNA methyltransferase genes and 4 DNA demethylase genes with 19 agronomic traits in 508 maize inbred lines. The *P*-value is shown on a −log_10_ scale (Bonferroni threshold, *P* < 1.22 × 10^−4^). **B)** Association of SNPs and InDels from *ZMET2* with the number of husk layer. InDel-7080 is most significantly associated variant with the number of husk layer based on an association assay with 201 SNPs/InDels. The *P*-value is shown on a −log_10_ scale (Bonferroni threshold, *P* < 2.46 × 10^−4^). The gene structure of *ZMET2* is shown at the bottom. UTRs, exons, and introns/promoter regions are shown as open boxes, filled boxes, and dark lines, respectively. The translation start codon (ATG) and stop codon (TAA) are shown on the panel, respectively. **C)** The pattern of pairwise linkage disequilibrium (LD) of the variations. **D)** Sequence alignment of the locus 7080 in the 3′UTR of *ZMET2* from Indel-7080In and Indel-7080Ref maize varieties. **E)** Boxplot showing the number of husk layer of each haplotype. The box shows the median, and the lower and upper quartiles, and the dots denote outliers. Statistical significance was determined by a Mann–Whitney *U* test, **P < 0.01.

To determine the probable contribution of genetic polymorphisms of DNA methylation–related genes to phenotypic diversity in maize, we performed an association study using 449 single-nucleotide polymorphisms (SNPs) spanning 7 DNA methyltransferase genes and 4 DNA demethylase genes with 19 agronomic traits in a maize association panel that consisted of 508 inbred lines (Yang et al. 2010; Li et al. 2013). The significant associations were only seen between 3 SNPs within *ZMET2* (*Zm00001d026291*) with the number of husk layers (*P* = 4.23 × 10^–5^; Fig. 1A; Supplementary Table S1).

To further elucidate the genetic basis by which *ZMET2* affects husk layer variation, we resequenced *ZMET2* from 200 randomly selected inbred lines. An 8.1-kb genomic region that spanned both the 5′UTR and 3′UTR of *ZMET2* was analyzed. A total of 177 SNPs and 24 Indels (minor allele frequency ≥ 0.05) were further identified. Under a mixed linear model (Yu et al. 2006), the 10-bp Indel-7080 within the 3′UTR showed the most significant association with husk layer diversity (*P* = 1.21 × 10^–4^; Fig. 1, B and C; Supplementary Table S2). Based on this variant, the 200 maize line genotypes were classified into 2 major haplotypes (Supplementary Table S3). The inbred lines represented by the B73 reference allele without the 10-bp insertion (Indel-7080Ref) had a significantly lower number of husk layers than the inbred lines with the 10-bp insertion (Indel-7080In, *P* < 0.001; Fig. 1, D and E).

### Variations in the 3′UTR alter *ZMET2* transcript and global DNA methylation levels

**Figure 2.**
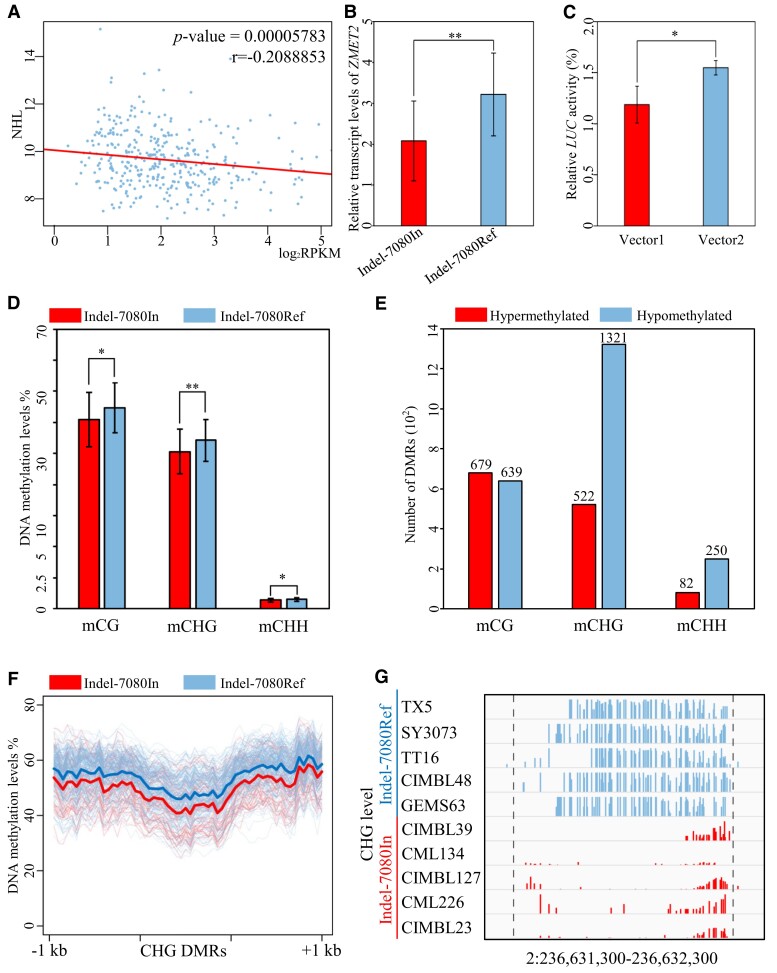
Genetic variations in *ZMET2* affect its transcript and DNA methylation. **A)** The expression level of *ZMET2* is correlated to the number of husk layer. Pearson correlation coefficient (*r*) and *P*-values are shown on the top right corner. **B)** Comparison of the *ZMET2* transcript levels among 12 Indel-7080In lines and 12 Indel-7080Ref lines. Error bars indicate the Sd of the mean. Mann–Whitney *U* test, **P < 0.01. **C)** The reporter construct of Indel-7080In significantly repressed LUC activity relative that with Indel-7080Ref. Error bars represent mean ± Sd from 5 replicates. Student's *t*-test, **P < 0.01. **D)** The average level of CG, CHG, and CHH methylation from the study by Xu et al. (2019) (*n* = 48 for lines with Indel-7080In; *n* = 182 for lines with Indel-7080Ref). Error bars are Sd, and significant differences are determined using the Mann–Whitney *U* test, *P < 0.05, **P < 0.01. **E)** Number of hypermethylation and hypomethylation for CG, CHG, and CHH DMRs between Indel-7080In lines and Indel-7080Ref lines. **F)** Metaplots showing CHG methylation across CHG DMRs. **G)** Examples of hypo-CHG DMRs. Each track represents different inbred lines. *x* axis indicates regions of DMR. *y* axis of each track indicates levels of CHG methylation (0% to 100%).

Given that Indel-7080 locates in the 3′UTR of *ZMET2*, we hypothesized that it might alter the transcript abundance of *ZMET2* and thereby contribute to husk layer variation among diverse inbred lines (Wang et al. 2006; Akdeli et al. 2014; Mauger et al. 2019). Based on RNA-sequencing (RNA-seq) data for the same association population (Fu et al. 2013), we detected a negative correlation between the *ZMET2* transcript level and the number of husk layers (*r* = −0.2088853, *P* = 5.78 × 10^–5^; Fig. 2A). In addition, we further compared the transcript level of *ZMET2* among 24 randomly selected inbred lines, which included 12 lines carrying Indel-7080Ref and 12 lines carrying Indel-7080In. Our real-time quantitative PCR (qPCR) analysis showed that the transcript abundance of *ZMET2* in lines with Indel-7080Ref was significantly higher than that in lines carrying Indel-7080In (Fig. 2B).

To further ascertain the effect of Indel-7080 on *ZMET2* transcript abundance, the 3′UTRs of *ZMET2* from Indel-7080Ref and Indel-7080In were cloned downstream of the luciferase (LUC) open reading frame to construct the reporter vectors *pGreen0800-mini promoter* + *LUC* + 3′-UTR–Indel-7080In (Vector I) and *pGreen0800-mini promoter* + *LUC* + 3′-UTR–Indel-7080Ref (Vector II) (Supplementary Fig. S1A). Maize mesophyll protoplasts were transfected with each recombinant LUC vector for transient expression assays. As shown in Fig. 2C, vectors fused with the 3′UTR containing Indel-7080Ref exhibited significantly higher LUC activity than those with Indel-7080In (Vector II vs. Vector I; Mann–Whitney *U* test, *P* = 0.016), and the transcription level showed significant differences (Supplementary Fig. S1B). Together, these results indicated that the Indel-7080 polymorphism in the 3′UTR of *ZMET2* was able to alter its transcript abundance.

Variation in *ZMET2* transcript abundance may consequently affect DNA methylation in the natural population. To test this possibility, we performed whole-genome bisulfite sequencing (WGBS) of 12 randomly selected inbred lines, 6 of which carried the Indel-7080Ref allele, whereas the other 6 carried the Indel-7080In allele (Supplementary Table S4). Ear samples at the early growing stage were collected for sequencing. As *ZMET2* is involved in non-CG methylation (Du et al. 2014; Li et al. 2014; Fu et al. 2018; Fang et al. 2022), we focused on CHG and CHH methylation in this analysis. In the context of CHG and CHH, we could not detect a statistically significant difference in the total level of DNA methylation between Indel-7080Ref and Indel-7080In lines, regardless of genic regions or transposable elements (Supplementary Fig. S2). We suspected that the absence of such differences was likely due to the limited number of samples in this analysis. To test this possibility, we compared DNA methylation patterns in 230 maize inbred lines (48 lines with Indel-7080In and 182 lines with Indel-7080Ref), which were previously generated using a capture-based method (Han et al. 2018; Xu et al. 2019). As expected, inbred lines with Indel-7080In had significantly lower levels of CHG methylation than lines with Indel-7080Ref (Mann–Whitney *U* test, *P* < 0.01; Fig. 2D). These results suggested that the variable *ZMET2* transcript abundance attributed to Indel-7080 might at least partly contribute to the natural variation in the global CHG methylation level in maize.

The differential DNA methylation levels between 2 *ZMET2* genotypes might enable specific effects in some methylated regions of the genome. To test this possibility, differentially methylated regions (DMRs) were identified for CG, CHG, and CHH contexts between Indel-7080In and Indel-7080Ref lines. In total, we identified 1,843 CHG DMRs. More than 71.7% of these CHG-DMRs (1,321/1,843) showed hypomethylation in Indel-7080In lines relative to Indel-7080Ref lines (Fig. 2, E and F). An example was shown in Fig. 2G, where a region with reduced DNA methylation in Indel-7080In compared to that in Indel-7080Ref lines. For CG DMRs, only 48.5% (639/1,318) showed hypomethylation. This suggested that the methylation differences in CG DMR may not be caused by variations in *ZMET2* but rather by the fact that the methylation data come from natural populations, where a large number of DMRs have already been identified in previous studies (Xu et al. 2019). There are relatively few CHH DMRs between Indel-7080Ref lines and Indel-7080In lines (Fig. 2E), indicating that the CHG methylation was sensitive to *ZMET2* compared to CHH methylation in the natural population. These results suggested that natural *ZMET2* variants not only affect the total level of CHG methylation but also modulate CHG methylation at thousands of sites in a locus-specific manner.

### *ZMET2* negatively regulates the number of husk layers

**Figure 3.**
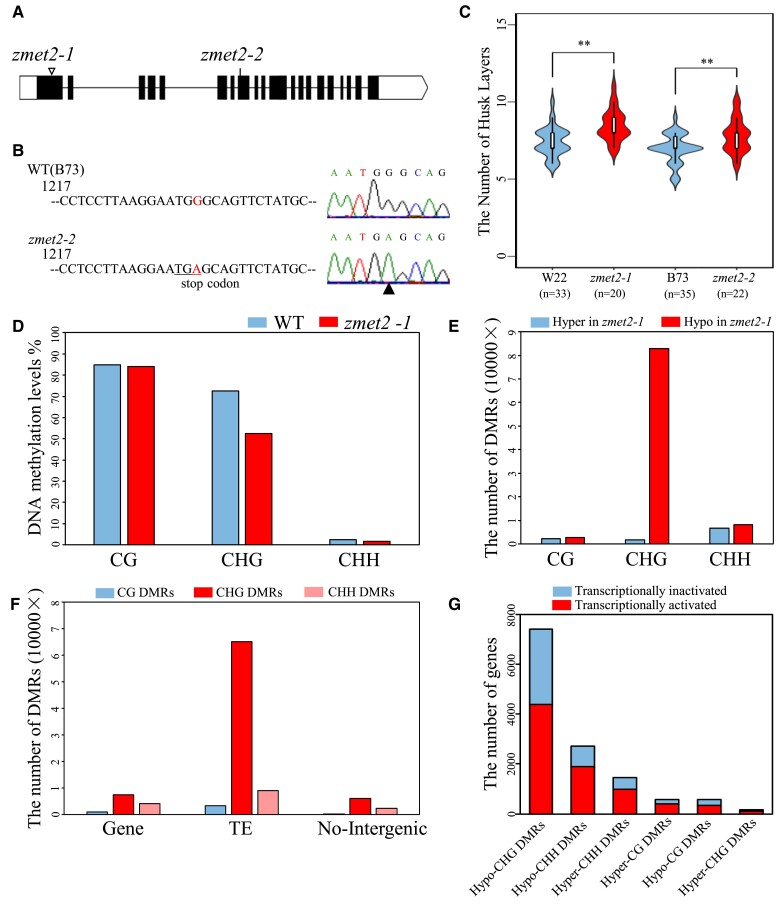
*ZMET2* regulates husk development and DNA methylation. **A)** Gene structure of *ZMET2* with mutation sites. The triangle in the exon 1 indicates the mutator insertion site in *zmet2–1*. Black and white boxes represent exons and untranslated regions, respectively. **B)** Sanger sequencing to show a single base mutation from **G)** to **A)** in exon 8 of *ZMET2*, leading to a premature stop coding. **C)** Violin plot showing the number of husk layer of each haplotype. The inner white box represents the interquartile range. The central line represents the median value. The outer shape indicates the kernel-density curve. Statistical significance was determined by the Mann–Whitney *U* test, **P < 0.01. **D)** The average level of global cytosine CG (left), CHG (middle), and CHH (right) methylation in WT and mutant plants. **E)** Number of hypomethylated DMRs or hypermethylated DMRs in the 3 different sequence contexts between WT and *zmet2-1* mutant. **F)** Number of hypomethylated DMRs or hypermethylated DMRs that overlapped with different genomic features. **G)** Number of genes with DMRs distribution; blue or red boxes indicate transcriptionally activated or transcriptionally inactivated genes. TE, transposable elements; WT, wild type.

To verify the role of *ZMET2* in regulating husk development, we analyzed a mutant allele of *ZMET2* caused by a Mu insertion in the W22 inbred background (*mu1013094*) from UniformMu stocks (www.maizeGDB.org), hereafter referred to as *zmet2-1* (Fig. 3A). This mutant allele has been used to characterize *ZMET2* function in previous studies (Li et al. 2014; Fu et al. 2018). A second mutant allele in the B73 inbred background (EMS4-24c1ab; hereafter, *zmet2-2*) was obtained from the Maize EMS-induced Mutant Database (Lu et al. 2018). Sequence analysis confirmed a single base mutation from G to A in the eighth exon of *zmet2-2*, which resulted in a premature stop codon (underlined TGA in Fig. 3B). Phenotypic investigation showed that the number of husk layers was significantly increased in both *zmet2-1* and *zmet2-2* homozygous mutants as compared with their corresponding wild-type plants (Fig. 3C). In addition, mutant plants exhibited fewer days to anthesis and reduced number of leaves above the primary ear and branches of tassel as compared with wild-type plants (Supplementary Fig. S3), whereas no significant differences were detected for total leaf number, plant height, hundred kernel weight, diameter of cob, and 2 tassel-related traits (Supplementary Fig. S3).

### *ZMET2* regulates CHG and CHH methylation

To examine the alteration in DNA methylation, we carried out a WGBS analysis on the developing ear with the husk layer primordium in *zmet2-1* mutant and wild-type plants. In the *zmet2-1* mutant, both CHG and CHH, but not CG methylation levels, were remarkably reduced (Fig. 3D), supporting the earlier conclusion that *ZMET2* is specifically required for CHG and CHH methylation.

To define *ZMET2*-mediated CHG and CHH methylation at a fine scale, DMRs were identified, yielding a total of 82,914 hypomethylated CHG DMRs and 8,134 hypomethylated CHH DMRs in the mutant as compared with wild-type plants (Fig. 3E). In contrast, only 1,691 hypermethylated CHG DMRs and 6,676 hypermethylated CHH DMRs were identified in the mutant (Fig. 3E). The *ZMET2*-sensitive DMRs were largely found in transposons because transposons make up a large proportion of the genome (Fig. 3F). However, hypomethylated CHG and CHH DMRs in the genic regions were associated with 7,223 genes, 66.9% of which were transcriptionally active (Fig. 3G). These results suggested that *ZMET2*-sensitive DMRs might regulate gene expression.

### *ZMET2* regulates gene expression by altering CHG and CHH methylation

To ascertain how the altered DNA methylation correlated with gene expression, RNA-seq experiments were conducted from the same samples that were used for methylation analysis (described above, Supplementary Table S5). A total of 2,203 differentially expressed genes (DEGs) (|log_2_(FC)| > 1, false discovery rate [FDR] < 0.05) were identified, including 1,512 upregulated and 691 downregulated DEGs in the *zmet2-1* mutant as compared with wild-type plants (Supplementary Table S6).

**Figure 4.**
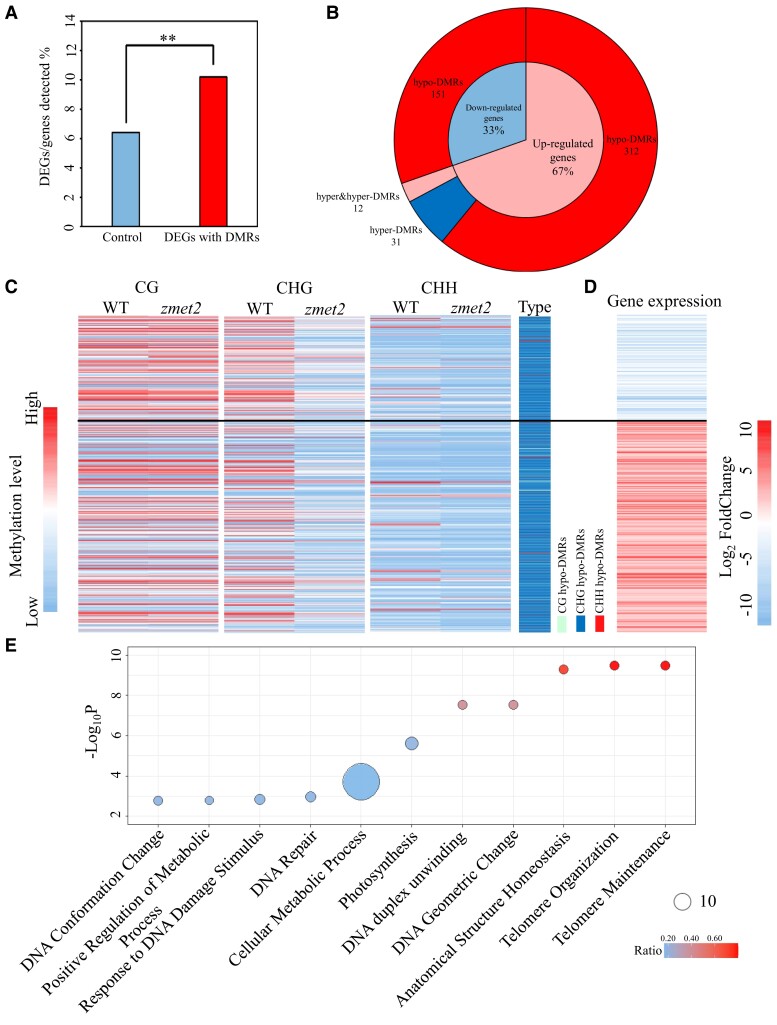
*ZMET2* regulates gene expression. **A)** Association of DEGs with DMRs. Statistical significance was determined by a prop.test, **P < 0.01. **B)** Pie charts showing DEGs associated with differentially DMRs. **C)** Heatmaps displaying changes in methylation of DEGs within hypomethylated DMRs in wild-type and *zmet2-1* mutant plants. Types of hypomethylated DMRs distributed in DEGs are shown on the right. **D)** Heatmaps showing the expression level of DEGs within hypomethylated DMRs in wild-type and *zmet2-1* mutant plants. The blocks represent log_2_(FC) of DEGs (FDR < 0.05, |log2(FC)| ≥ 1). **E)** GO enrichment analysis of upregulated DEGs within hypomethylated CHG DMRs. Each bubble represents 1 functional class, and the size of the bubble indicates the number of enriched genes within each GO term. The *y* axis shows the *P*-values from Fisher test.

Next, we examined the impact of changes in DNA methylation on the transcription of genes associated with DMRs. The identified hypomethylated CHG and CHH DMRs were associated with 4,835 expressed genes (i.e. expressed genes and promoter regions within a DMR), and 10.1% of these genes were identified as DEGs, which was significantly higher than the genome-wide average (7.8%, prop.test, *P* < 0.01; Fig. 4A). DEGs were more likely to be associated with hypomethylated DMRs (82%) than with hypermethylated DMRs (18%). In addition, 67% of DEGs associated with hypomethylated DMRs showed upregulation, whereas the remaining 33% were downregulated (Fig. 4B). Heatmaps established connections between the methylome and transcriptome levels of DEGs associated with hypomethylated DMRs (Fig. 4, C and D). The increased proportion of DEGs that were associated with hypomethylated DMRs suggested that the altered DNA methylation was directly relevant to gene expression in the *zmet2-1* mutant.

Gene Ontology (GO) analyses indicated that only upregulated genes within hypomethylated CHG DMRs (genes within 2 kb of a DMR) were enriched for multiple biological processes, including telomere maintenance and organization, anatomical structure homeostasis, DNA geometric change, DNA duplex unwinding, photosynthesis, cellular metabolic process, DNA repair, response to DNA damage stimulus, positive regulation of the metabolic process, DNA conformation change, and others (Fig. 4E; Supplementary Table S7). These results suggested that transcriptional changes in a substantial number of genes could contribute to the altered number of husk layers in *zmet2* mutants.

### Indel-7080 was targeted by selection

**Figure 5.**
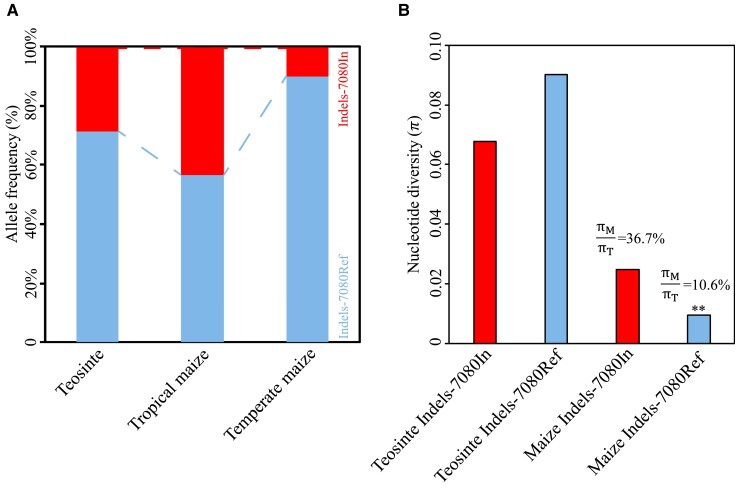
Indel-7080 is targeted by selection. **A)** The allele frequency of Indel-7080 in teosinte, tropical maize, and temperate maize inbred lines. **B)** Nucleotide diversity analysis of the region surrounding Indel-7080 in teosinte and maize. $\pi_{M}$/ $\pi_{T}$ indicates the amount of nucleotide diversity (*π*) retained in maize relative to that in teosinte. *P*-values were determined using coalescence simulations, **P < 0.01.

To examine the evolutionary origin of Indel-7080, we sequenced the flanking region of Indel-7080 in 21 diverse teosinte accessions (*Z. mays* ssp. *parviglumis*) and 181 maize inbred lines. The results showed that 28.6% and 71.4% of teosinte accessions carried the Indel-7080In and Indel-7080Ref allele, respectively (Fig. 5A; Supplementary Table S8), indicating that Indel-7080 is a standing genetic variant in teosinte. The Indel-7080Ref allele, with its associated decrease in husk layers, was present in 56.8% of tropical maize inbred lines but rose to 90.2% of temperate maize inbred lines (Fig. 5A).

To examine whether Indel-7080 has been subjected to selection, we analyzed nucleotide diversity surrounding Indel-7080 in maize and teosinte (Beissinger et al. 2016). The maize lines containing the Indel-7080In allele retained 36.7% of the nucleotide diversity present in teosinte, whereas the nucleotide diversity of maize lines containing the Indel-7080Ref allele retained only 10.6% of the diversity of teosinte (Fig. 5B). Further coalescence simulation incorporating the maize domestication bottleneck revealed that the Indel-7080Ref allele had undergone intensive selection (Fig. 5B). These results suggested that the Indel-7080Ref allele played different roles during maize domestication and adaptation, ultimately leading to the rapid accumulation of the less-husk allele in temperate maize germplasm.

## Discussion

DNA methylation varies across the genome, as well as among individuals in many taxa, and functions as an epigenetic regulator of gene expression (Zhang et al. 2006; Eichten et al. 2013; Kawakatsu et al. 2016). A great deal of general information has been published concerning molecular pathways or regulatory genes involved in establishing and maintaining DNA methylation (Zhang et al. 2018; He and Feng 2022; Sun et al. 2022), but almost nothing is known about how epigenetic alterations contribute to natural phenotypic variation in maize. Our data demonstrated that genetic polymorphisms in the DNA CMT gene *ZEMT2* led to natural variation in the number of husk layers by modulating CHG and CHH methylation.

Our GWAS (Genome-wide association studies), which involved integrating 11 DNA methylation–related genes and 19 agronomic traits, showed that only genetic polymorphisms in *ZMET2* were significantly associated with natural variation in the phenotypic diversity represented by the number of husk layers in a diverse maize association population. This raises an intriguing question: Why were no other DNA methylation–related genes detected in this analysis? This might be easily explained by the low power of GWAS in general to detect those cases in which rare alleles, rather than common alleles, exist for other genes that explain the variance among some inbred lines (Pritchard 2001; McCarthy and Hirschhorn 2008; Uffelmann et al. 2021). Alternatively, although multiple agronomic phenotypes were assessed in this study, we cannot rule out the possibility that natural polymorphisms in these DNA methylation-related genes contribute to other aspects of maize growth and development. Future extensive surveys of additional phenotypic traits would help determine whether this possibility is true.

In *zmet2* mutants, in addition to the effect on the number of husk layers, several other morphological changes were also observed, including days to anthesis, the number of leaves above the ear and branches of tassel. We note that none of these phenotypes in *zmet2* mutants were reported in previous studies (Papa et al. 2001; Cao et al. 2003; Xu et al. 2019; Uffelmann et al. 2021). We suspect that the mild alterations noted for these phenotypes may render them easy to be missed in the observation. However, it is not clear why only the number of husk layers, but not the other traits, such as days to anthesis, which is also included in this study, was associated with *ZMET2* variants in our natural association population. There are 2 possible explanations for this phenomenon. First, compared with artificial mutants, natural alleles often affect gene function to a lesser extent. Indeed, we have demonstrated that a 10-bp Indel in the 3′UTR of *ZMET2* resulted in polymorphisms by influencing its transcriptional abundance. Therefore, it is easily understandable that the natural *ZMET2* alleles have minor effects on gene function relative to the 2 *zmet2* mutants used in the study. In this scenario, the different developmental stages may need a certain threshold of ZMET2 activity, in which the formation of husk layers is more sensitive to *ZMET2* dosage than other traits. Second, although specific DNA methyltransferases may have unique targets, there is evidence that the partial-to-complete redundancy of different DNA methyltransferases for establishing and maintaining the DNA methylation pattern occurs in a locus- or tissue-specific manner (Li et al. 2014; Gouil and Baulcombe 2016; Zhao et al. 2022). In this context, apart from its effect on husk layers, ZMET2 may act redundantly with other MET, CMT, or DRM methyltransferases in controlling other aspects of plant growth and development.

In the *zmet2-1* mutant, 84,605 CHG DMRs and 14,810 CHH DMRs were identified, and the majority of these DMRs were hypomethylated, supporting the previous conclusion that *ZMET2* is critically required for CHG and CHH methylation in maize (Papa et al. 2001; Li et al. 2014; Gouil and Baulcombe 2016; Fu et al. 2018; Zhao et al. 2022). Interestingly, a large number of DMRs could be identified between 2 *ZMET2* genotypes in the natural population, indicating that the genetic variance of *ZMET2* creates naturally occurring epigenetic variation in maize. This is consistent with an early study showing that *ZMET2* is involved in the maintenance of epigenetic states that show natural variation between the B73 and Mo17 inbred lines (Makarevitch et al. 2007). Hence, our results further demonstrate the existence of *ZMET2*-mediated natural epigenetic variation in exceedingly more diverse inbred lines.

Naturally occurring epigenetic variation contributes to phenotypic variation and the adaptability of plants under various environmental conditions. Among teosinte accessions, alleles with the more-husk Indel-7080In or less-husk Indel-7080Ref have been diversified. Our findings that the frequency of Indel-7080Ref varied across teosinte-to-tropical and teosinte-to-temperate inbred lines suggested that Indel-7080 might have undergone different selective pressures during maize domestication and adaptation. This raises an intriguing question of whether the selection of Indel-7080 directly benefits maize domestication from teosinte and the later spread of maize from tropical to temperate zones. Modern maize varieties grown in tropical areas often require heavy husk coverage to protect the ear from pathogens, whereas maize varieties planted in northern temperate regions often have reduced husk coverage to allow faster ear dry-down, which is beneficial for mechanical harvesting (Glenn et al. 2017). Therefore, we speculated that the Indel-7080Ref might play a role in the spread of maize in temperate regions, which in turn conferred advantages for maize growth and production. However, the possibility that *ZMET2* has been selected as a result of changes in other traits but with a correlation with husk layer development cannot be ruled out and awaits further detailed exploration.

## Materials and methods

### Plant materials

Mutator mutant lines in the maize (*Z. mays*) W22 inbred background, *mu1013094*, were obtained from UniformMu stocks, and 1 EMS allele (*zmet2-2*, EMS4-24c1ab) was obtained from the Maize EMS Induced Mutant Database (MEMD, http://www.elabcaas.cn/memd/), which contains 1 nonsense mutation substitution in the coding region of the gene.

### Resequencing the *ZMET2* region

According to the B73 reference sequence (B73 RefGen_v4) (Jiao et al. 2017), 7 pairs of primers (Supplementary Table S9) were used to sequence an 8.1-kb region around *ZMET2*, including coding sequence, upstream and downstream sequence, in a diverse maize panel containing 200 inbred lines (Supplementary Table S3). These sequences were assembled and aligned by MEGA version 7 (http://megasoftware.net/). Polymorphic sites (SNPs and InDels) were identified, and their association with the number of husk layers and the levels of LD between sites were calculated using TASSEL (Bradbury et al. 2007).

### qPCR

Total RNA was extracted from developing ears (<0.5 cm in length) with TRIzol (Invitrogen) as per the manufacturer's instructions and then reverse transcribed into cDNA with the PrimeScript II 1st Strand cDNA Synthesis Kit (6210A; Takara, Shiga, Japan). Real-time qPCR analysis was performed on a Bio-Rad CFX96 Real-Time PCR Detection System using the 2× TB Green Premix Ex Taq II Kit (Takara). The comparative C_T_ (2^−ΔΔCT^) method (Schmittgen and Livak 2008) was used to quantify the different mRNA levels. Three biological replicates and 3 technical replicates for each biological replicate were used for each primer combination, and sample type. *ZmUBQ1* (*Zm00001d019684*) was used as the reference gene. Sequences of primers used in qPCR are listed in Supplementary Table S9.

### Protoplast transient expression assays

A minimal promoter from the cauliflower mosaic virus (mpCaMV) was inserted upstream of the firefly LUC coding sequence in pGreenII 0800-LUC, a commercial dual-LUC assay vector, to drive the expression of the LUC reporter gene. In the same construct, a Renilla (REN) LUC reporter gene under the control of the 35S promoter was used as an internal control to evaluate the protoplast transfection efficiency. The 3′UTR from B73 that differed only at the Indel-7080 site was amplified by PCR (Supplementary Table S9). The PCR products and the SacII-digested (New England Biolabs) pGreenII 0800-LUC, which share an identical 19-bp sequence, were mixed to allow site-specific recombination using a Hieff Clone One-step PCR Cloning Kit (Yeasen Biotechnology). This generated vectors *pGreen0800-mini promoter* + *LUC* + 3′-UTR–Indel-7080Ref (Vector I) and *pGreen0800-mini promoter* + *LUC* + 3′-UTR–Indel-7080In (Vector II). Both vectors were confirmed by sequencing prior to their use in the transient expression assays. Maize mesophyll protoplast isolation and subsequent transfection were performed as described (Huang et al. 2018). LUC and REN activities were assayed using a Dual-LUC Reporter Assay System (Promega) according to the standard protocol. Relative LUC activity was calculated by normalizing LUC to REN activity (LUC/REN). Five biological replicates, each with 2 technical replicates, were assayed per vector.

### Library preparation and WGBS

In brief, genomic DNA was isolated from the developing ear (<0.5 cm in length). Samples were fragmented and ligated with TruSeq-methylated adapters. Bisulfite conversion was performed on 500 ng of adaptor-ligated DNA using the MethylCode Bisulfite Conversion Kit (Zymo Research, Orange, CA, USA). The converted DNA was amplified for 14 cycles. Libraries were paired-end sequenced on the Illumina Nova 6000 platform. Sequencing reads (GEO accession GSE232004; Supplementary Table S4) were processed to identify and filter poor-quality sequences and incomplete conversions. Sequences were aligned to the B73 reference genome (AGPv4) using the Bismark aligner (v0.24.0) under the parameters (-N 1) (Krueger and Andrews 2011; Jiao et al. 2017). Methylated cytosines were extracted from aligned reads using the Bismark methylation extractor under standard parameters.

### RNA-seq and differential gene expression analysis

Total RNA was extracted from developing ears (<0.5 cm in length) with TRIzol (Invitrogen) as per the manufacturer's instructions. RNA-seq libraries were generated using the NEBNext Ultra II RNA Library Prep Kit (model no. E7770S; New England Biolabs). The libraries were sequenced using the HiSeq 150-bp paired-end Illumina RNA-seq on the Nova6000 platform. The generated raw data have been deposited in the National Center for Biotechnology Information's Gene Expression Omnibus database repository (http://www.ncbi.nlm.nih.gov/geo/) under accession number GSE232004. Details about deposited data files are listed in Supplementary Table S5. Raw sequencing reads were first processed with Trimmomatic to remove low-quality bases at the 5′ and 3′ ends (*q* < 20) (Bolger et al. 2014), and reads > 30 bp were used for mapping to the B73 reference sequence v4 (AGPv4) by using HISAT2 v2.2.0 (Pertea et al. 2016). Unique mapping reads were considered for mRNA-level quantification using HTSeq-count v0.12.4 with the intersection-strict option (Supplementary Table S3) (Anders et al. 2015). DEGs were identified using the DEseq2 package and nonnormalized raw count data (Love et al. 2014). Differences in expression were considered statistically significant with a FDR ≤ 0.05 and |log_2_(FC)| > 1. We conducted GO term analysis on selected gene lists using AgriGO2.0 with default parameters (http://systemsbiology.cau.edu.cn/agriGOv2/). We used a significance cutoff at FDR < 0.05.

### Identification of DMRs

The DMRs between Indel-7080In and Indel-7080Ref lines in the natural population were identified using the following method. Only the cytosines covered by at least 2 reads were considered for further analysis. Each maize genome was divided into nonoverlapping 100-bp windows. The windows meeting the following criteria were kept: More than 50% of lines in both haplotypes had at least 3 cytosine sites for CG or CHG within the window. More than 50% of lines in both haplotypes had at least 6 methylation sites for CHH within the window. The retained adjacent windows were merged into DNA methylation regions using BEDTools (Quinlan and Hall 2010), and the DNA methylation level of each region was recalculated for each line. A *t*-test was performed on each DNA methylation region between 2 haplotypes. The CG or CHG differential methylation regions (DMRs) were defined as regions with a *P*-value of <0.05 and a difference greater than 10%, while the CHH DMRs were defined as regions with a *P*-value of <0.05 and a difference greater than 5%.

To identify DMRs between *zmet2-1* and wild type, the maize genome was first divided into consecutive nonoverlapping 100-bp windows. Next, the number of cytosine sites and the average coverage for each sequence context (CG, CHG, or CHH) within each window were calculated for each sample. The 100-bp windows with at least 2× coverage that had at least 3 cytosine sites for CG or CHG and 6 cytosine sites for CHH were retained. The average DNA methylation level of these remaining windows was calculated. Windows were kept if the difference in the level of DNA methylation between wild-type and mutant plants was ≥60% for CG and CHG. For CHH, the windows that met the following criteria were kept: >20% difference between 2 genotypes, with 1 having <5% methylation and the other having >25% methylation. Finally, the adjacent windows were merged, and the DNA methylation levels of these merged regions were calculated. DEGs associated with different categories of DMRs were the genes within the corresponding category of DMRs (CHG or CHH context) in the promoter region (2-kb upstream region of gene transcript) or the gene body (containing 5′UTR and 3′UTR).

### Nucleotide diversity analysis and selection test

A 360-bp fragment encompassing *ZMET2* InDel7080 was investigated in 21 teosinte accessions (*Z. mays* ssp. *parviglumis*) (Supplementary Table S8). The primers used to amplify the region are listed in Supplementary Table S9. Nucleotide diversity (*π*) was calculated using a 100-bp sliding window with a 25-bp step using the software DnaSP (version 6.12.03) (Librado and Rozas 2009). The amount of nucleotide diversity retained in maize relative to that in teosinte was calculated as the relative ratio of *π* in maize to *π* in teosinte. Coalescent simulations incorporating the domestication bottleneck were performed for the sequenced region to test whether the loss of genetic diversity in maize relative to that in teosinte was attributable to domestication selection using the Hudson's ms program (Hudson 2002). All parameters set in the analysis have been described previously (Tian et al. 2009; Huang et al. 2018). The population mutation and population recombination parameters were estimated from the teosinte data. A significant deviation from the expectation under a neutral domestication bottleneck indicated that the region tested was likely targeted by selection during maize domestication.

### Accession numbers

Sequence data from this article can be found in the GenBank data libraries under accession numbers GSE232004.

## Supplementary Material

kiae113_Supplementary_Data

## Supplementary data

The following materials are available in the online version of this article.

**Supplementary Figure S1.** Indel-7080 polymorphism in the 3′UTR of *ZMET2* alters *ZMET2* transcript.

**Supplementary Figure S2.** Comparisons of the average level of CHG and CHH methylation between Indel-7080Ref and Indel-7080In lines.

**Supplementary Figure S3.** Violin plots showing the 9 agronomic traits of each haplotype in wild-type and *zmet2-1* plants.

**Supplementary Table S1.** Association of SNPs in DNA methyltransferase genes and DNA demethylase genes with 19 agronomy traits in 508 maize inbred lines.

**Supplementary Table S2.** Association variants across the 8.1-kb sequenced region around ZMET2.

**Supplementary Table S3.** Information on the maize association panel used in this study.

**Supplementary Table S4.** BS-seq data summary and mapping results.

**Supplementary Table S5.** RNA-seq data summary and mapping results.

**Supplementary Table S6.** DEGs in zmet2-1 mutant as compared with wild-type plants.

**Supplementary Table S7.** GO term enrichment analysis in Fig. 4E.

**Supplementary Table S8.** The 21 diverse teosinte accessions used for molecular evolution analysis in this study.

**Supplementary Table S9.** Primers used in this study.

## Dive Curated Terms

The following phenotypic, genotypic, and functional terms are of significance to the work described in this paper:

CMT2 Gramene: AT4G19020
CMT2 Araport: AT4G19020

ZMET2 Gramene: Zm00001d026291
ZMET2 Araport: Zm00001d026291
