# Supplementary material for: Natural polymorphisms in ZMET2 encoding a DNA methyltransferase modulate the number of husk layers in maize
Source: Plant Physiol. 2024 Mar 2;195(3):2129–42. doi: 10.1093/plphys/kiae113 (PMC11213254; doi:10.1093/plphys/kiae113)
Supplement: kiae113_Supplementary_Data [file kiae113_supplementary_data.zip › Supplementary Figure.pdf]

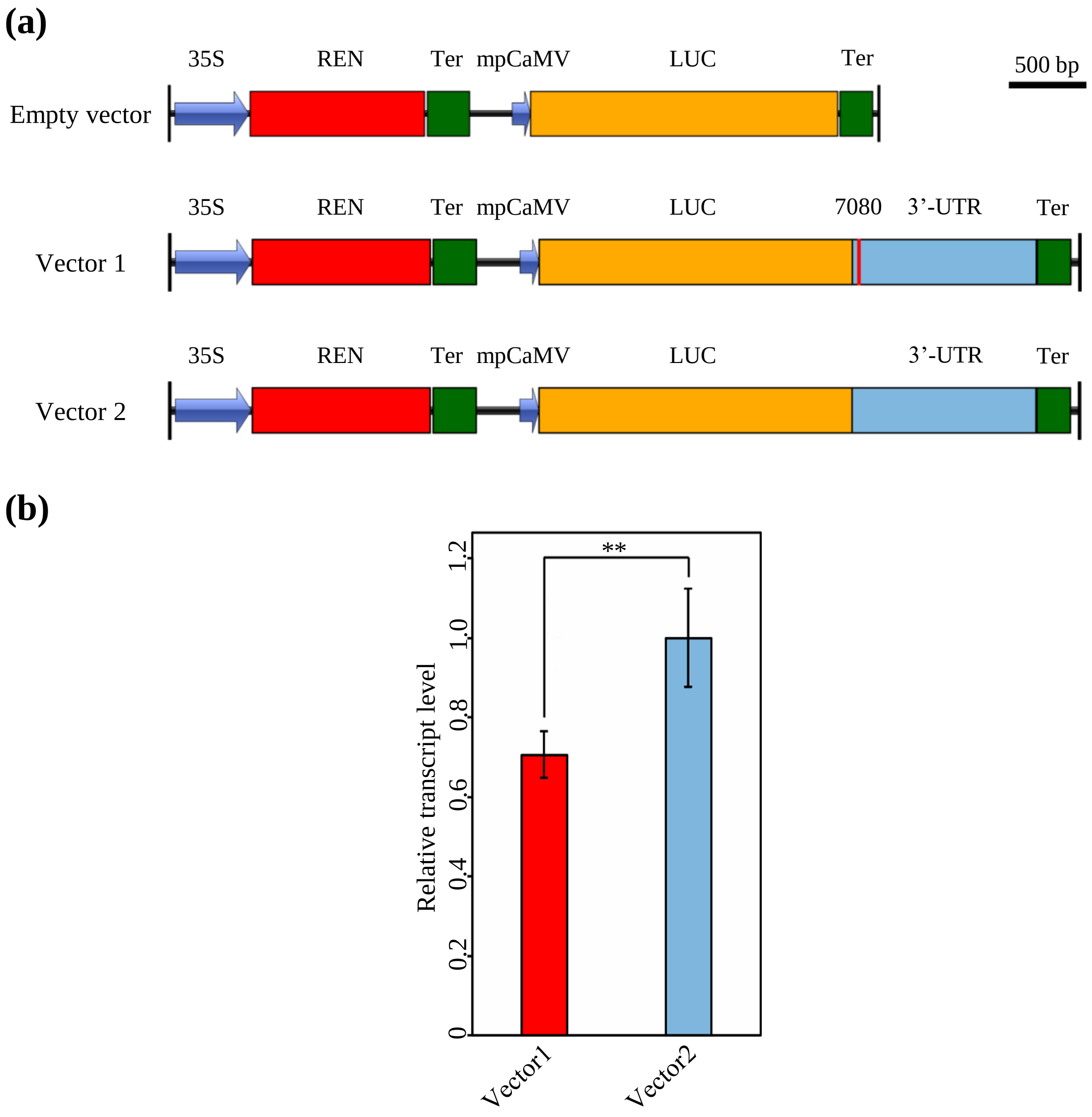

Supplemental Figure S1. Indel-7080 polymorphism in the 3'UTR of *ZMET2* alter *ZMET2* transcript. A. Vector constructs used to test the effects of the Indel-7080 variant in the 3'UTR of *ZMET2* in two haplotypes on gene expression during transient expression. **B.** The transcript levels of LUC (firefly luciferase) in Vector1 and Vector2. Error bars represent mean ± SD from five replicates. Statistical significance was determined by the t-test, ** P<0.01.

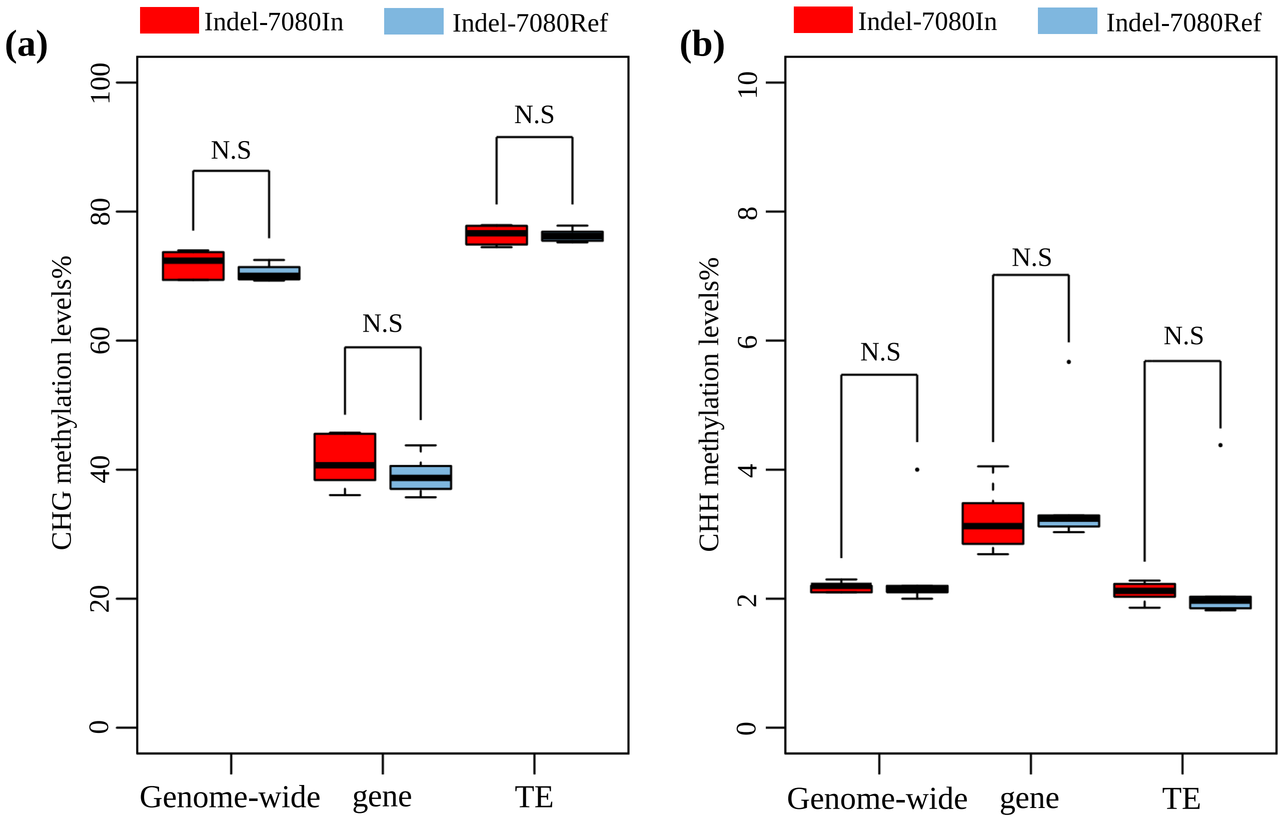

Supplemental Figure S2. Comparisons of the average level of CHG and CHH methylation between Indel-7080Ref and Indel-7080In lines. A, The average level of CHG in genome, genic regions and transposable elements (TE). **B,** The average level of CHH in genome, genic regions and transposable elements. n = 6 for lines with Indel-7080In, n = 6 for lines with Indel-7080Ref. The box in this figure shows the median, and the lower and upper quartiles, and the dots denote outliers. All significant differences in this figure are determined using the MannWhitney U -test, N.S., not significant (P＞0.01).

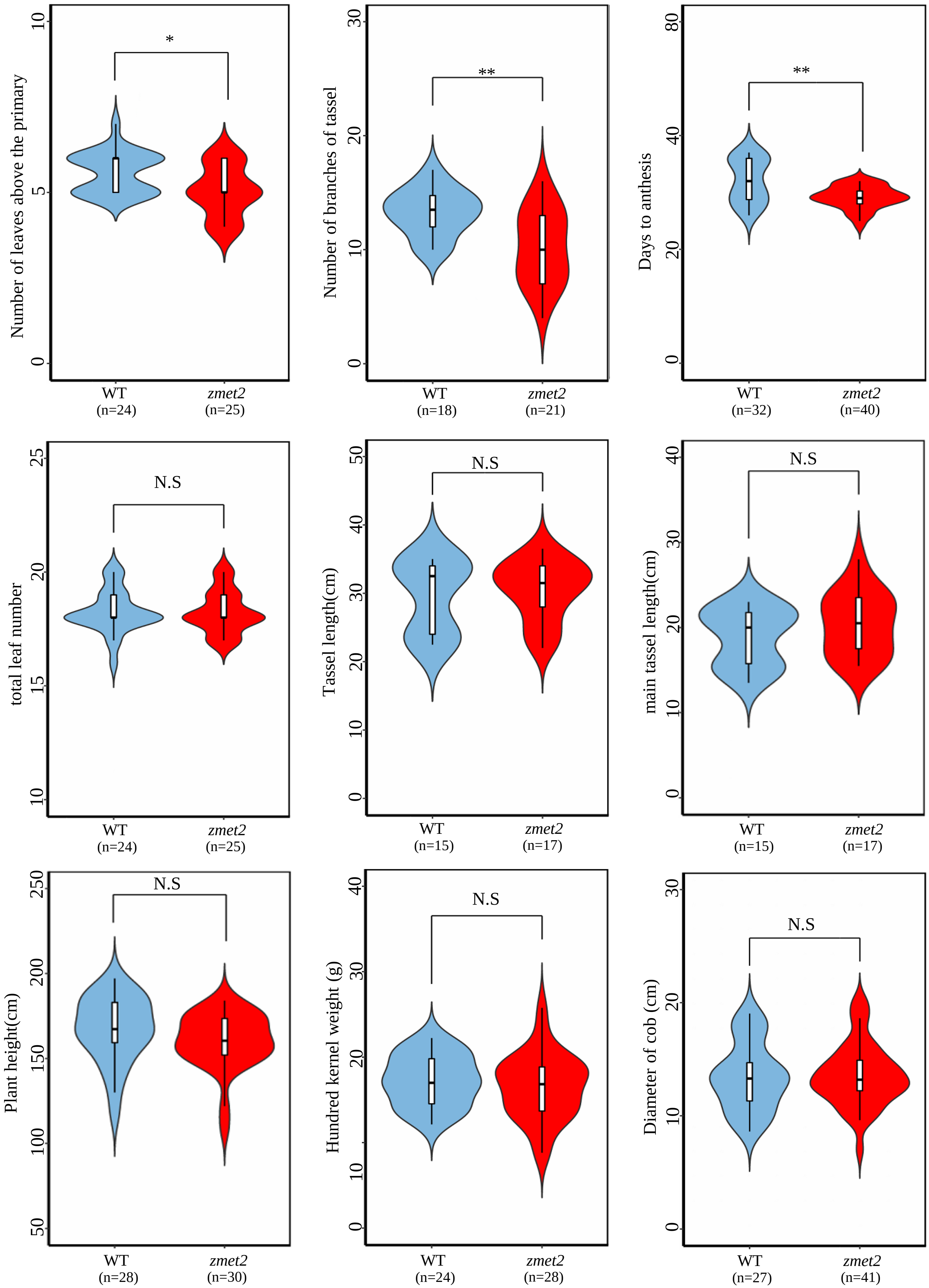
*
Number of leaves above the primary
WT (n=24)
zmet2 (n=25)
**
Number of branches of tassel
WT (n=18)
zmet2 (n=21)
**
Days to anthesis
WT (n=32)
zmet2 (n=40)
N.S
total leaf number
WT (n=24)
zmet2 (n=25)
N.S
Tassel length(cm)
WT (n=15)
zmet2 (n=17)
N.S
main tassel length(cm)
WT (n=15)
zmet2 (n=17)
N.S
Plant height(cm)
WT (n=28)
zmet2 (n=30)
N.S
Hundred kernel weight (g)
WT (n=24)
zmet2 (n=28)
N.S
Diameter of cob (cm)
WT (n=27)
zmet2 (n=41)

Supplemental Figure S3. Violin plots showing the nine agronomic traits of each haplotype in wild-type (WT) and *zmet2-1* plants. The inner red box represents the interquartile range. The central black line represents the median value. The outer gray shape on each side represents all measured data points and the thickness represents the probability density of the data. Violin plots with three lines, at median and 25th and 75th percentile and violin shape indicates the kernel-density curve. Only Statistical significance was determined by the MannWhitney U -test, * P<0.05, ** P<0.01, N.S., not significant (P＞0.01).
