# Supplementary material for: Natural polymorphisms in ZMET2 encoding a DNA methyltransferase modulate the number of husk layers in maize
Source: Plant Physiol. 2024 Mar 2;195(3):2129–42. doi: 10.1093/plphys/kiae113 (PMC11213254; doi:10.1093/plphys/kiae113)
Supplement: kiae113_Supplementary_Data [file pp.01336.2023-s04.pdf › pp.01336.2023-s04.pdf]

Plant Physiology®

Natural polymorphisms in ZMET2 encoding a DNA methyltransferase modulate the number of husk layers in maize

Zi Wang, Aiai Xia, Qi Wang, Zhenhai Cui, Ming Lu, Yushen Ye, Yanbo Wang, and Yan He

Review Timeline:

Submission Date:	29-Aug-2023
Editorial Decision:	24-Oct-2023
Revision Received:	30-Jan-2024
Accepted:	08-Feb-2024

24-Oct-2023

Prof. Dr. Yan He
China Agricultural University
Beijing 100083
China

RE: Natural polymorphisms in ZMET2 encoding a DNA methyltransferase modulate the number of husk layers in maize

Dear Dr. He:

We have received reviews of your manuscript, which you may view at the end of this letter. On the basis of the advice received and my own evaluation, I believe that, pending revision, this article may ultimately be acceptable for publication in Plant Physiology.

In particular, please pay attention to a few issues:
1) Detailed proofread and revision of the manuscript is needed, particularly, two reviewers pointed out quite a few places need to be rephrased.
2) Further analysis of the data is necessary, for example, reviewer 1 suggested in the second and fourth comments, comment 3 raised by reviewer 2.
3) As the first reviewer pointed out in the third comment, "These results are not sufficient for supporting the idea that the reduced expression of ZMET2 in the Indel-7080In varieties is involved in modulating the number of husk layers.", please clarify this either via experiment or explain somehow.

When you are ready to submit your revision, click the below link and find the red arrow under your Author Tasks heading for this revision submission.

If you want to visit this version of the manuscript, you may click the link below and then click on the folder that reads "Post Decision Manuscripts".

Link Not Available

When uploading the revised version of this article, please be sure to include as complete an answer as possible to the referees' criticisms during the first round of reviews in the "response to reviewers" section.

Plant Physiology considers a revision for up to 60 days after a manuscript has been provisionally accepted; limited extension of this timeframe is at the discretion of the Monitoring Editor. A reminder notice will be sent to you 2 weeks before the deadline, at which time you may choose to contact the editorial office to request an extension; if so, please provide a justification for your request. After the expiration of this deadline, the article would need to be submitted as a resubmission and would be handled as such.

Thank you very much for giving us an opportunity to review this work. I look forward to receiving the next version.

Sincerely,

Plant Physiology Board of Editors

Want to add this revision deadline to your calendar? Click below!

--- Reviewer comments:
Reviewer #1 (Comments for the Author):

The study identified a natural indel variation within the 3'UTR of ZMET2 in maize and reported that the polymorphism is

associated with the number of husk layers. Moreover, the study showed that the expression level of ZMET2 was lower in Indel-7080In varieties than in Indel-7080Ref varieties. Therefore, the study concluded that the higher number of husk layers is attributed to the lower expression level of ZMET2 in the Indel-7080In varieties. It is interesting to find that a natural variation in ZMET2 is associated with the number of husk layers in maize. However, whether the variation has a causative effect on the expression of ZMET2 and on the number of husk layers needs to be validated by more evidences.

1. In line 163-165, the statement was not correct. Based on the results, I feel that the sentence should change to "In the context of CHG and CHH, we could not detect statistically significant difference in the total level of ....".

2. Although ZMET2 was thought to regulate CHG and CHH methylation, the effect of Indel-7080 on DNA methylation should be analyzed not only at CHG and CHH sites but also at CG sites (in Figure 2d and 2e). The analysis of CG methylation can be used as a negative control in the results. The analysis will contribute to excluding the possibility that the weak difference in CHG and CHH methylation between indel-7080In and Indel-7080Ref was caused by experiment errors.

3. Two independent zmet2 mutant alleles were obtained to determine the effect of zmet2 on the number of husk layers. The increased number of husk layers was indeed observed in the zmet2 mutants relative to the wild-type controls (Figure 3c). However, in addition to increased number of husk layers, multiple other development defects were also observed in the zmet2 mutants (Supplemental Figure 3). These results are not sufficient for supporting the idea that the reduced expression of ZMET2 in the Indel-7080In varieties is involved in modulating the number of husk layers. Either a ZMET2 RNA interference lines or a CRISPR-induced deletion at 3'UTR should be obtained to confirm that the variation in 3'UTR of ZMET2 has a causative effect on the number of husk layers.

4. From Figure 3g, the authors suggest that most of genes with DMRs are transcriptionally active. In general, the DNA methylation at the promoter region was thought to repress gene expression. Therefore, it is necessary to compare the ratio of actively transcribed genes to random genes and the ratio of actively transcribed genes to DMR-containing genes. This is necessary to determine the transcription levels of genes with ZMET2-mediated methylation. Moreover, the DMRs in the promoter region and the gene body should be separately analyzed when determining their effects on gene expression.

5. In line 234 and 235, the text indicated that "DEGs were more likely to be associated with hypermethylated DMRs (18%)." This statement is not appropriate, because the number of hypo-DMRs is significantly higher than the number of hyper-DMRs at the whole-genome level (Figure 3e). The DMRs are not enriched more in DEGs than random.

6. The effects of zmet2 on DNA methylation and gene expression were combinatorially analyzed in Figure 4a-4d. Based on the results, the text indicated that the increased proportion of DEGs that were associated with hypomethylated DMRs. Although up-regulated genes contain a high portion of hypo-DMRs, as indicated in the main text, the down-regulated genes also contain a high portion of hypo-DMRs (Figure 4b). It is difficult to determine how ZMET2-dependent DNA methylation influences gene expression.

Reviewer #2 (Comments for the Author):

Comments:
This study reveals the innovative exploration of natural polymorphisms in DNA methylation-related genes and their impact on maize phenotypic variation. Identifying a 10-bp Indel variant in 3' UTR of ZMET2, altering transcript levels, and influencing husk layer numbers, provides valuable insights. Additionally, population genetic analyses highlight ZMET2's selection and potential role in maize spread. However, I still have some suggestions for this manuscript.

Major:
1. Page5, line 118, the threshold of P-value 1.22x10-4 is used. Could there be false negatives for SNPs? (Such as the P-value of SNP is 5x10-3) Is it possible that some SNPs/Indels that are relevant to the trait have been discarded?
2. Page5, line 125-126, "the 10-bp Indel-7080 within the 3'UTR showed the most significant association with husk layer diversity"; How are the correlations of the three SNPs mentioned above (line 118)? Since ZMET2 were selected based on these three SNPs, I am also interested in their individual performances.
3. Page 6, line 163-169, Supp-Fig 2 and Fig 2D. Using the 230 maize inbred lines to compare DNA methylation patterns is more rigorous, but why the trend of CHG between Indel-7080In and Indel-7080Ref seem to turnover in two analyses? When comparing with the 12 randomly selected inbred lines Indel-7080In seems CHG-hypermethylated, but the 230 maize inbred lines seem CHG-hypomethylated. I think it is better to proof that this is indeed a random event via Bootstrap's statistical method.
4. Page9, lines 232, "In addition, 66% of DEGs associated with hypomethylated DMRs showed upregulation, whereas the remaining 33% were downregulated": The descriptions about how the authors carried out related analyses were not made in necessary detail. Because of the DNA methylation of the gene body, the promoter region has different effects on the differential expression of genes, which is a little ambiguous here.

Minor:

1. Page 5, line 130, Figure legend 1E. Please give the full name in the Figure legend of this abbreviation "NHL" (in the Y-axis of Figure1.e). Is "number of husk layers"? And please check the full manuscript about this type of problem.
2. Page 5, line 133-135, "we hypothesized that it might alter transcript abundance of ZMET2 and thereby contribute to husk layer variation among diverse inbred lines". Please quote some references for this sentence.
3. Page 5, line 136-137, "significantly negative correlation between the ZMET2 transcript level and the number of husk layers (r = -0.2088853, P = 5.78 × 10-5". The P-value of r is lower than 0.01, but the absolute value of r is smaller than 0.25, which may mean no relationship between variables. It is better to describe this result with more appropriate words.
4. Page 8, line 213, Figure 3e. Same as the minor-1, please check it.
5. Page 15, line 409. In line 222, |log2(FC)| > 2 was used, but here the threshold is 1. Please check it.

Reviewer #1

The study identified a natural indel variation within the 3'UTR of ZMET2 in maize and reported that the polymorphism is associated with the number of husk layers. Moreover, the study showed that the expression level of ZMET2 was lower in Indel-7080In varieties than in Indel-7080Ref varieties. Therefore, the study concluded that the higher number of husk layers is attributed to the lower expression level of ZMET2 in the Indel-7080In varieties. It is interesting to find that a natural variation in ZMET2 is associated with the number of husk layers in maize. However, whether the variation has a causative effect on the expression of ZMET2 and on the number of husk layers needs to be validated by more evidences.

Thank you for your nice comments about our work. Following your suggestions, we have added the corresponding data and made correction on several errors in the revised manuscript. The detailed point-by-point responses are listed below.

1. In line 163-165, the statement was not correct. Based on the results, I feel that the sentence should change to "In the context of CHG and CHH, we could not detect statistically significant difference in the total level of ....".

RESPONSE. Thank you for pointing it out. We have corrected this error in the revised manuscript. Please see page 6, line 162.

2. Although ZMET2 was thought to regulate CHG and CHH methylation, the effect of Indel-7080 on DNA methylation should be analyzed not only at CHG and CHH sites but also at CG sites (in Figure 2d and 2e). The analysis of CG methylation can be used as a negative control in the results. The analysis will contribute to excluding the possibility that the weak difference in CHG and CHH methylation between indel-7080In and Indel-7080Ref was caused by experiment errors.

RESPONSE. Thank you for the great suggestion. Inbred lines with Indel-7080In had significantly lower levels of CHG methylation than lines with Indel-7080Ref ($P < 0.01$). In contrast, the differences of CG and CHH methylation ($0.01< P < 0.05$) were not as significant as CHG methylation (Figure 2D). In addition, a total of 1,318 CG DMRs, 1,843 CHG DMRs and 332 CHH DMRs were identified between Indel-7080In and Indel-7080Ref lines. Over 71.7% of CHG-DMRs (1,321/1,843) showed hypomethylation in Indel-7080In lines relative to Indel-7080Ref lines. However, for CG-DMRs, only 48.5% (639/1,318) showed hypomethylation. This suggested that the methylation differences in CG-DMR may not be caused by variations in *ZMET2*, but rather by the fact that the methylation data come from natural populations, where a large number of DMRs have already been identified in previous studies (Xu et al., 2019; Xu et al., 2020). Meanwhile, relatively few CHH DMRs indicated that CHH methylation was not sensitive to *ZMET2* compared to CHG methylation in the natural population. These analyses suggested that the difference in CHG methylation between indel-7080In and Indel-7080Ref was more than likely caused by the variations of *ZMET2*.

3. Two independent zmet2 mutant alleles were obtained to determine the effect of zmet2 on the number of husk layers. The increased number of husk layers was indeed observed in the zmet2 mutants relative to the wild-type controls (Figure 3c). However, in addition to increased number of husk layers, multiple other development defects were also observed in the zmet2 mutants (Supplemental Figure 3). These results are not sufficient for supporting the idea that the reduced

expression of ZMET2 in the Indel-7080In varieties is involved in modulating the number of husk layers. Either a ZMET2 RNA interference lines or a CRISPR-induced deletion at 3'UTR should be obtained to confirm that the variation in 3'UTR of ZMET2 has a causative effect on the number of husk layers.

RESPONSE. Here we determined the effect of *ZMET2* on the number of husk layers using two independent mutants in the different inbred backgrounds. In fact, the *ZMET2* gene in the inbred line ND101 which is able to transgene in our transformation facility harbors 3'UTR without Indel-7080, therefore it is impractical to create a CRISPR-induced deletion targeting Indel-7080. However, fortunately, the *zmet2-1*, a Mu insertion in the W22 inbred background and widely used previous studies, is a knockdown mutant as the qRT-PCR analysis showed that the expression *ZMET2* is significantly decreased but not abolished in *zmet2-1* plants (Figure a, below). In addition, a significant difference in the expression of *ZMET2* (Figure 2b) and in the number of husk layers (Figure b, below) has been demonstrated in the 24 random-selected inbreeding for indel-7080In and Indel-7080Ref lines. Moreover, vectors fused with the 3'UTR containing Indel-7080Ref exhibited significantly higher luciferase activity and transcription level than those with Indel-7080In (the new Supplemental Figure 1B). Together, these experiments indicated that the variation in 3'UTR of *ZMET2* has a causative effect on the number of husk layers.

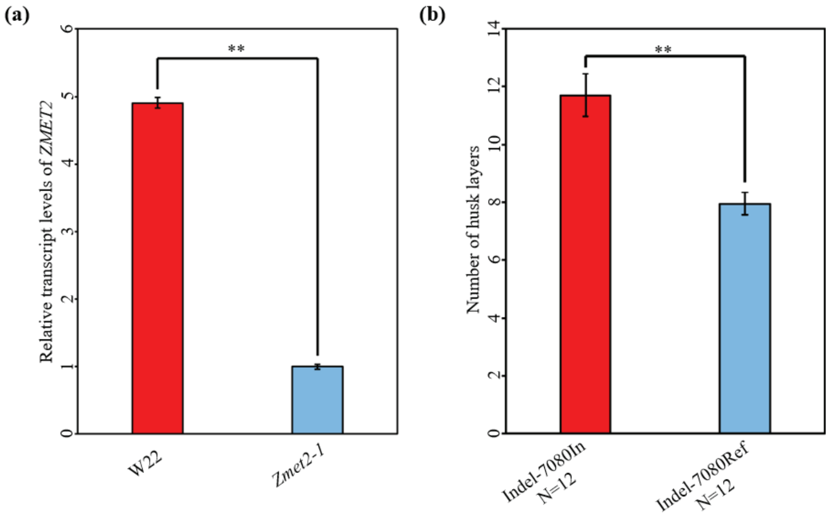

4. From Figure 3g, the authors suggest that most of genes with DMRs are transcriptionally active. In general, the DNA methylation at the promoter region was thought to repress gene expression. Therefore, it is necessary to compare the ratio of actively transcribed genes to random genes and the ratio of actively transcribed genes to DMR-containing genes. This is necessary to determine the transcription levels of genes with ZMET2-mediated methylation. Moreover, the DMRs in the promoter region and the gene body should be separately analyzed when determining their effects on gene expression.

RESPONSE. Thank you for the great suggestion. As suggested, we have considered the diversity of DMRs in the promoter region (2kb upstream region of gene transcript) and the gene body, and then compared the ratio of active transcription genes to random genes, as well as the ratio of active transcription genes containing CHG-hypo DMRs to genes with

CHG-hypo DMRs distribution. The blue boxes indicate the ratio of active transcription genes to random genes; the middle box indicate the ratio of active transcription genes with CHG-hypo DMRs distributed in promoter region to all genes with CHG-hypo DMRs distributed in promoter region, and the right box indicate the ratio when CHG-hypo DMR distributed in gene body (Figure below). The proportion of active transcription genes with DMR in the promoter region (70.6%) and gene body (68.6%) is higher than the whole genome level (58.9%). These results suggested that *ZMET2*-sensitive DMRs are involved in the regulation of gene expression.

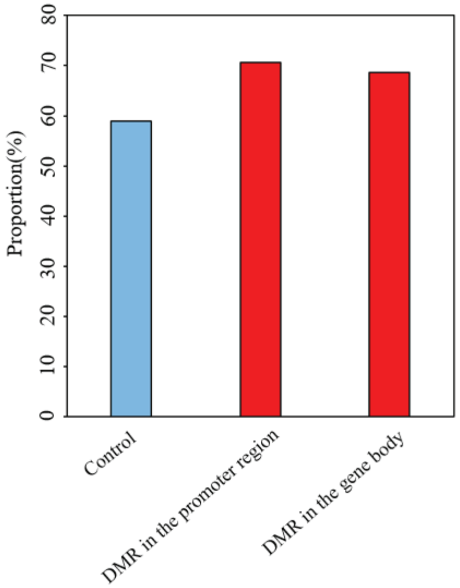

5. In line 234 and 235, the text indicated that "DEGs were more likely to be associated with hypermethylated DMRs (18%)." This statement is not appropriate, because the number of hypo-DMRs is significantly higher than the number of hyper-DMRs at the whole-genome level (Figure 3e). The DMRs are not enriched more in DEGs than random.

RESPONSE. We apologized for unclear on it. The statement in the text is: "DEGs were more likely to be associated with hypomethylated DMRs (82%) than with hypermethylated DMRs (18%)." There are more hypomethylated DMRs distributions in DEGs. Following your suggestion in the previous question, we focus on genes within hypo-CHG DMRs and hypo-CHH DMRs distributed in promoter regions and gene bodies. The enrichment degree of these DMRs in DEGs is higher than that of random ones (Figure 4a).

6. The effects of zmet2 on DNA methylation and gene expression were combinatorially analyzed in Figure 4a-4d. Based on the results, the text indicated that the increased proportion of DEGs that were associated with hypomethylated DMRs. Although up-regulated genes contain a high portion of hypo-DMRs, as indicated in the main text, the down-regulated genes also contain a high portion of hypo-DMRs (Figure 4b). It is difficult to determine how ZMET2-dependent DNA methylation influences gene expression.

RESPONSE. Thank you for the great suggestion. DEGs exhibited much higher proportion of association with hypomethylated DMRs (82%) than with hypermethylated DMRs (18%). In

addition, 67% of DEGs associated with hypomethylated DMRs showed upregulation (Figure 4B). The reason why a portion of the down-regulated genes with DMRs could be explained by the possibility as below. Gene-associated DNA methylation in plants can occur in the promoter or within the transcribed gene body. Promoter DNA methylation usually inhibits gene transcription, although in some cases it promotes gene transcription(Zhang et al., 2018). Promoter DNA methylation directly represses transcription by inhibiting the binding of transcription activators or promoting the binding of transcription repressors or indirectly represses transcription by promoting repressive histone modifications such as H3K9me2 and inhibiting permissive histone modifications such as histone acetylation (Domcke et al., 2015; Possomato-Vieira, José S. and Khalil and Modeling, 2017). Presumably, DNA methylation may enhance the binding of some transcription activators or may inhibit the binding of some transcription repressors. In the case of genes activated by promoter DNA methylation, active demethylation causes transcriptional silencing of the gene(Lei et al., 2015; Lang et al., 2017). This issue involves the study of downstream candidate genes, which is also our next research direction.

Reviewer #2

This study reveals the innovative exploration of natural polymorphisms in DNA methylation-related genes and their impact on maize phenotypic variation. Identifying a 10-bp Indel variant in 3' UTR of ZMET2, altering transcript levels, and influencing husk layer numbers, provides valuable insights. Additionally, population genetic analyses highlight ZMET2's selection and potential role in maize spread. However, I still have some suggestions for this manuscript.

Thank you for your nice comments about our work. Following your suggestions, we have added the corresponding data and made correction on several errors in the revised manuscript. The detailed point-by-point responses are listed below.

Major:

1. Page5, line 118, the threshold of P-value 1.22×10^{-4} is used. Could there be false negatives for SNPs? (Such as the P-value of SNP is 5×10^{-3}) Is it possible that some SNPs/Indels that are relevant to the trait have been discarded?

RESPONSE. Thank you for the great point. Our P-value is derived from Bonferroni-adjusted threshold, which is a reliable method used in numerous recent studies (Li et al., 2013; Zhang et al., 2019; Li et al., 2022). In addition, we fully agreed that although the false negatives likely happen under such stringent threshold, it would be precise to obtain the real SNPs by avoiding false positives.

2. Page5, line 125-126, "the 10-bp Indel-7080 within the 3'UTR showed the most significant association with husk layer diversity"; How are the correlations of the three SNPs mentioned above (line 118)? Since ZMET2 were selected based on these three SNPs, I am also interested in their individual performances.

RESPONSE. The two SNPs located on the last exon are synonymous mutations with a p-value of 5.13×10^{-3} and 4.82×10^{-3}, respectively. The other SNP locates on the eighth intron with a p-value of 7.66×10^{-3}.

3. Page 6, line 163-169, Supp-Fig 2 and Fig 2D. Using the 230 maize inbred lines to compare DNA methylation patterns is more rigorous, but why the trend of CHG between Indel-7080In and Indel-7080Ref seem to turnover in two analyses? When comparing with the 12 randomly selected inbred lines Indel-7080In seems CHG-hypermethylated, but the 230 maize inbred lines seem CHG-hypomethylated. I think it is better to proof that this is indeed a random event via Bootstrap's statistical method.
RESPONSE. We apologized for unclear on it. Although the trend of DNA methylation in Indel-7080In seemed slightly higher than Indel-7080Ref in 12 randomly selected lines, we could not detect statistically significant difference in the DNA methylation between them (Supplemental Figure 2). We suspected that the absence of such differences was likely due to the limited number of samples in this analysis. As expected, in larger sample analysis, inbred lines with Indel-7080In had significantly lower levels of CHG methylation than lines with Indel-7080Ref (Figure 2D). In addition, the date in Supplemental Figure 2 was analyzed by whole-genome bisulfite sequencing (WGBS) of 12 randomly selected inbred lines, whereas the data source in Figure 2D was from a capture-based method in previous Research (Xu et al., 2019). The capture space includes over 20,000 regions, covering 15 Mb of the maize genome (Han et al., 2018). These regions were selected based on previous work of DNA methylation in maize and include regions that vary in DNA methylation across three maize lines or five maize tissues, mCHH islands, and promoters of genes that are potentially silenced by DNA methylation (Li et al., 2015a; Li et al., 2015b). The level of CHG methylation in the Figure 2D was not at the whole genome level, so it is lower than the level in Supplemental Figure 2. Considering that two dataset cover completely different methylated genomic regions, it is impractical to perform Bootstrap's statistical method.

4. Page9, lines 232, "In addition, 66% of DEGs associated with hypomethylated DMRs showed upregulation, whereas the remaining 33% were downregulated": The descriptions about how the authors carried out related analyses were not made in necessary detail. Because of the DNA methylation of the gene body, the promoter region has different effects on the differential expression of genes, which is a little ambiguous here.
RESPONSE. Thank you for pointing it out. Both DEGs within DMRs in the promoter region (2kb upstream region of gene transcript) and DEGs within DMRs in the gene body (contain 5'UTR and 3'UTR regions) tend to upregulate expression in *zmet2-1* (Figure below). Therefore, we analyzed these two types of DMRs together. DEGs associated with hypomethylated DMRs were the genes within hypomethylated DMRs (CHG or CHH context) in the promoter region (2kb upstream region of gene transcript) or the gene body (contain 5'UTR and 3'UTR regions). DEGs associated with hypermethylated DMRs were analyzed by the same method. We have added details related to our analysis in the Materials and Methods section. Please see page 16, line 442-445.

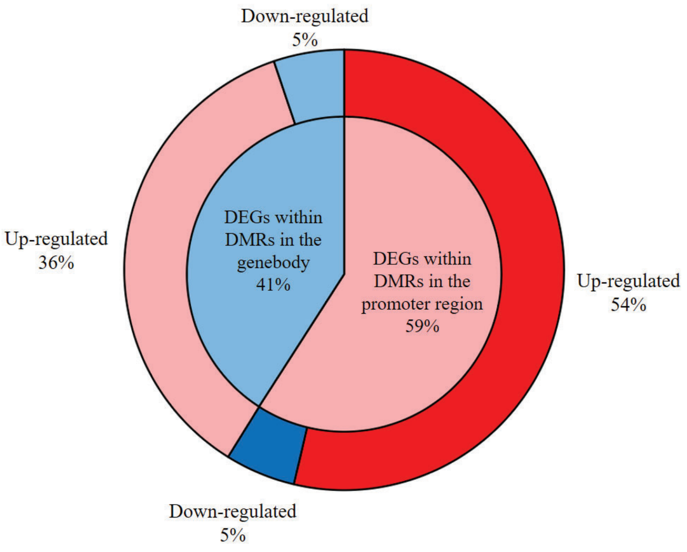

Minor:

1. Page 5, line 130, Figure legend 1E. Please give the full name in the Figure legend of this abbreviation "NHL" (in the Y-axis of Figure1.e). Is "number of husk layers"? And please check the full manuscript about this type of problem.

RESPONSE. Thank you. We have corrected it throughout the manuscript.

2. Page 5, line 133-135, "we hypothesized that it might alter transcript abundance of ZMET2 and thereby contribute to husk layer variation among diverse inbred lines". Please quote some references for this sentence.

RESPONSE. Thank you. We have added the related references for this sentence.

3. Page 5, line 136-137, "significantly negative correlation between the ZMET2 transcript level and the number of husk layers (r = -0.2088853, P = 5.78 × 10-5". The P-value of r is lower than 0.01, but the absolute value of r is smaller than 0.25, which may mean no relationship between variables. It is better to describe this result with more appropriate words.

RESPONSE. Thank you. We have amended this sentence this revised manuscript. Please see page 5, line 136-138.

4. Page 8, line 213, Figure 3e. Same as the minor-1, please check it.

RESPONSE. Thank you. We have corrected it this revised manuscript.

5. Page 15, line 409. In line 222, |log2(FC)| > 2 was used, but here the threshold is 1. Please check it.

RESPONSE. We have corrected it into $|\log_2(FC)| \geq 1$ in the revised manuscript.

08-Feb-2024
Prof. Dr. Yan He
China Agricultural University
Beijing 100083
China

MSID: PP2023-RA-01336R1
MS TITLE: Natural polymorphisms in ZMET2 encoding a DNA methyltransferase modulate the number of husk layers in maize

Dear Dr. Yan He:

I am pleased to inform you that your revised manuscript "Natural polymorphisms in ZMET2 encoding a DNA methyltransferase modulate the number of husk layers in maize" has been accepted for publication in Plant Physiology. Your manuscript will be checked for consistency with journal formatting and image standards by one of our Science Editors, who will contact you shortly. They may have a number of queries and suggestions for you. Please keep in mind that they work to increase the online visibility of your article and its attraction for readers from the widest of backgrounds. You will have the opportunity to review any revisions, and I hope you will work constructively with them to get the most out of your research. You will also have the opportunity to review all changes when you receive the author proofs for your article.

Once validated for publication, your article will be sent to a Science Editor for a final review of your manuscript and source files. After your final files are uploaded, your article will appear in the next available issue and the online Preview version of your article will be posted after your signed Copyright and Checklist forms and final source files are received.

Please be sure to include the following in your manuscript file:
Harvard style citations
List of all author contributions and funding information at the beginning of manuscript after the titles and author list.
List all supplemental materials associated with the article, with titles, at the end of the ms, before the references and acknowledgments section.
Complete Figure Legends for all figures appearing in the article listed before the Literature Cited.

Thank you and congratulations on your Plant Physiology paper!

Sincerely,
Plant Phys Board of Editors
Plant Physiology

=====
FIRST AUTHOR PROFILES
=====

We recognize the hard work that goes into being First Author of a Plant Physiology paper by publishing a "First Author Feature" for each First Author alongside your paper.

If the first author(s) would like to submit information for a feature on Facebook and Twitter, fill out the form by clicking on the link below:

Plant Physiology First Author Feature Form: https://forms.office.com/r/ZBCGZfhm8F
If you cannot access the form through the link above, please contact Rachel at rbelsky@aspb.org.

To upload an image of yourself to accompany your profile, please put your photo in the correct journal folder at the link below. You must label your image: Author First Name Last Name Article Title.

First Author Profile Image Dropbox: https://www.dropbox.com/scl/fo/xz029p7qjr2jml2rp2kmq/h?dl=0&rlkey=6fkxcaqmxx35ap152b2osy1fz

=====
IMPORTANT REMINDER: PEER REVIEW REPORTS

=====

If you opted to publish a peer review report along with your article during the original submission process, it will be prepared by the editorial staff and publicly posted with your manuscript, inside the zip file that contains any other supplemental material. As a reminder, the peer review report is a public record of all comments from editors and reviewers, as well as your prior responses, as you received them in the decision letters for each draft of your manuscript. If you agreed to publish this report and have changed your mind, or are not sure if you selected this option, please contact the editorial office as soon as possible before signing the license agreement from our publisher.

-- Reviewer comments:
Reviewer #1 (Comments for the Author):

My comments on the first version have been adressed by further analyses or experiments in the current version manscript. The study reported natural polymorphisms in the maintenance DNA methyltransferase gene of maize and demonstrated their effects on DNA methyltion and development. The study will conribute to understanding the mechanisms of DNA methylation and development in maize.

Reviewer #2 (Comments for the Author):

The authors have successfully addressed all of my comments.
